# Case Report: Acquired CDKN2A/B deletion and mTOR inhibitor resistance in TSC2-mutant metastatic epithelioid angiomyolipoma

**DOI:** 10.3389/fmed.2026.1832914

**Published:** 2026-08-25

**Authors:** Shuai He, Jing Yang, Jiguang Yang, Chunxia Li, Juan Shen, Xiaolong Ye, Zuping Liu, Wenhao Shen, Jun Zheng, Yongquan Wang

**Affiliations:** 1 Department of Urology, The First Affiliated Hospital (Southwest Hospital) of Army Medical University, Chongqing, China; 2 Institute of Urology, The First Affiliated Hospital (Southwest Hospital) of Army Medical University, Chongqing, China; 3 International Center for Aging and Cancer, Hainan Medical University, Haikou, Hainan, China; 4 Department of Colorectal and Urology Surgery, Gucheng County Hospital of Traditional Chinese Medicine, Xiangyang, Hubei, China; 5 Department of Pathology, The First Affiliated Hospital (Southwest Hospital) of Army Medical University, Chongqing, China

**Keywords:** acquired resistance, case report, CDKN2A/B, epithelioid angiomyolipoma, everolimus, metastatic renal tumor, TSC2 mutation

## Abstract

Acquired resistance to mTOR inhibition remains a major challenge in TSC2-mutant metastatic epithelioid angiomyolipoma (EAML). We report a 30-year-old woman with metastatic EAML harboring biallelic TSC2 mutations who achieved a complete response to everolimus but developed disease progression after 16 months. Tumor rebiopsy revealed persistent TSC2 mutations and acquired CDKN2A/B deletion, accompanied by increased Ki-67 and emergence of TFE-3 immunoreactivity and focal S-100 positivity. These findings suggest a potential association between CDKN2A/B loss and aggressive tumor behavior after mTOR inhibition, although causality remains unproven. CDK4/6-targeted strategies may warrant further investigation in this context.

## Key points

- TSC2-mutant metastatic EAML may initially respond well to mTOR inhibition but can develop acquired resistance during follow-up.
- Radiographic progression on mTOR inhibition should prompt repeat biopsy and molecular profiling when feasible.
- In this case, resistance was associated with CDKN2A/B homozygous deletion, persistent TSC2 alterations, increased proliferative activity (Ki-67 10% → 40%), and histological evolution.
- CDKN2A/B loss may be associated with mTOR inhibitor resistance and represents a potential biomarker for further investigation in combination therapeutic strategies.

## Introduction

Epithelioid angiomyolipoma (EAML) is a rare and potentially malignant variant of angiomyolipoma within the family of perivascular epithelioid cell tumors (PEComas) (1–3). It is characterized by a predominance of epithelioid cell morphology and exhibits clinically aggressive behavior, including local recurrence and distant metastasis. While EAML may occur sporadically, it is frequently association with tuberous sclerosis complex (TSC), highlighting a central molecular mechanism in its pathogenesis. Inactivating mutations in TSC1 or TSC2 result in constitutive activation of the mTOR signaling pathway, which plays a key role in tumor development (4–6). Diagnosis is based on characteristic histomorphology and is supported by immunohistochemical positivity for HMB-45 and Melan-A, which are essential for distinguishing EAML from other renal neoplasms (7, 8).

For localized disease, complete surgical resection remains the primary curative approach. However, therapeutic options for unresectable or metastatic PEComas remain limited, reflecting the rarity and biological heterogeneity of these tumors (9). Historically, systemic treatments including cytotoxic chemotherapy and anti-angiogenic agents have shown variable and generally modest efficacy, although occasional responses have been reported. Advances in molecular characterization have established aberrant activation of the TSC1/TSC2–mTOR pathway as a defining oncogenic event in a substantial proportion of PEComas, providing a strong rationale for pathway-directed therapy (10). Accordingly, mTOR inhibitors have emerged as the cornerstone of systemic treatment, with everolimus demonstrating clinical benefit in advanced PEComas. More recently, nab-sirolimus, an albumin-bound formulation of sirolimus, has shown promising antitumor activity in patients with malignant PEComas and represents an important therapeutic advancement in this disease setting (11, 12). Nevertheless, both intrinsic and acquired resistance to mTOR inhibition remain major challenges, and the molecular mechanisms driving treatment failure remain poorly understood.

Here, we report a case of metastatic TSC2-mutant EAML with initial response to everolimus followed by acquired resistance. Longitudinal molecular analysis identified CDKN2A/B deletion during disease progression, suggesting a potential link between cell cycle dysregulation and therapeutic resistance.

## Case presentation

### Patient information and initial presentation

**Figure 1:**
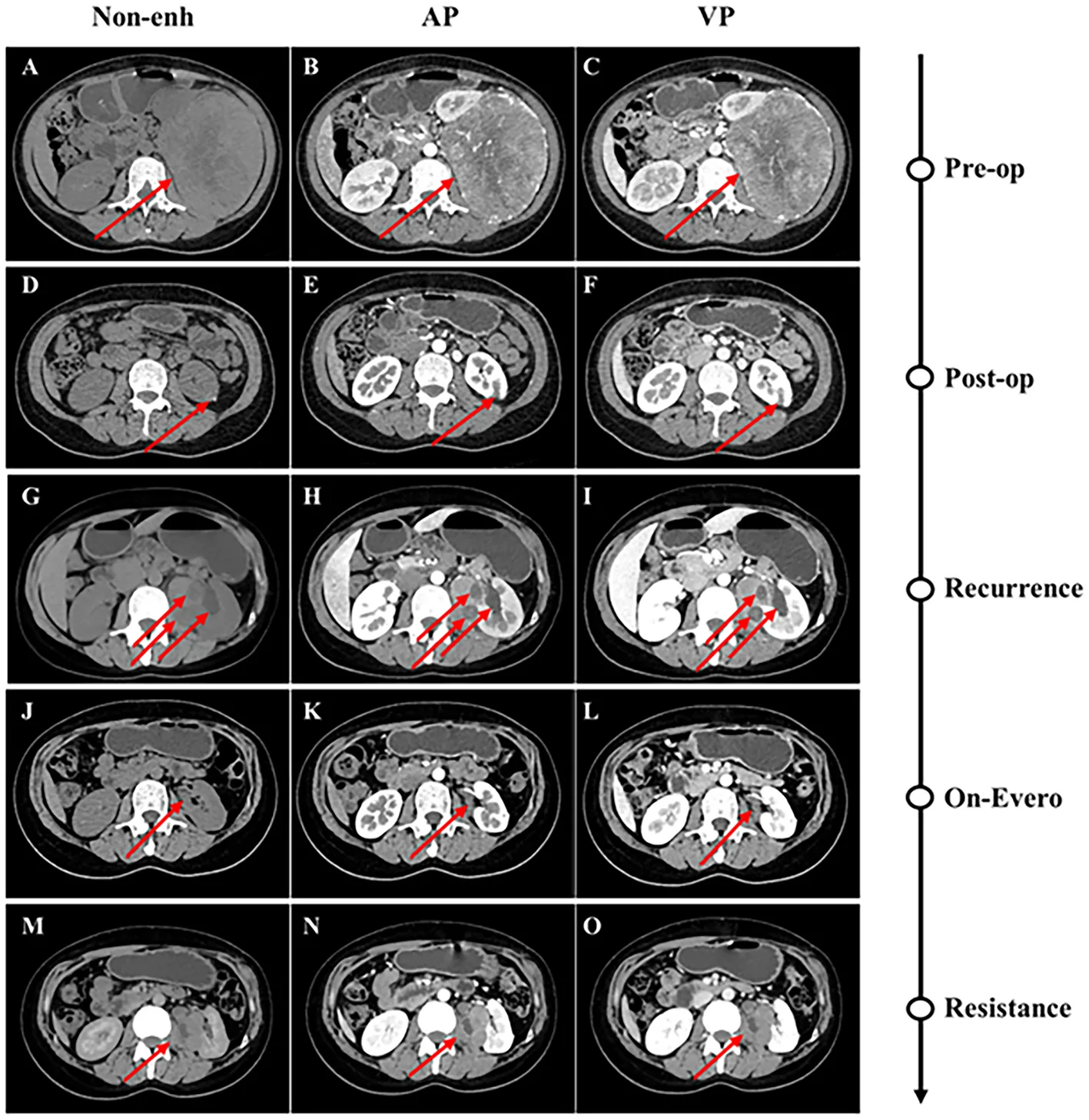
Serial axial contrast-enhanced CT imaging demonstrates the clinical course of metastatic epithelioid angiomyolipoma (EAML). **(A–C)** Baseline imaging reveals a heterogeneously enhancing left renal mass (arrows), with subsequent CT-guided biopsy confirming EAML. **(D–F)** Postoperative imaging following left partial nephrectomy shows complete tumor resection without residual disease. **(G–I)** Surveillance CT at 10 months demonstrates local recurrence in the surgical bed (arrows). **(J–L)** After initiation of everolimus, follow-up imaging show complete radiographic response. **(M–O)** Disease progression with new hepatic metastases at 16 months indicates acquired resistance to mTOR inhibition. AP, arterial phase; VP, venous phase; Non-enh, non-enhanced.

A 30-year-old woman was referred to our department for evaluation of an incidentally detected left renal mass identified three months earlier (Figures 1A–C and Supplementary Figures 1A–F). CT-guided percutaneous biopsy was consistent with EAML. During this period, the patient reported an unintentional weight loss of 4 kg, while remaining hemodynamically stable. Her medical history was unremarkable except for a previous cesarean section. No personal or family history of TSC, renal tumors, PEComa-associated disease, or hereditary cancer syndromes were reported. No psychosocial factors relevant to diagnosis, treatment selection, or adherence were identified. On physical examination, the patient was afebrile and hemodynamically stable, with no flank tenderness, palpable abdominal mass, or gross hematuria.

### Treatment and pathologic findings

**Figure 2:**
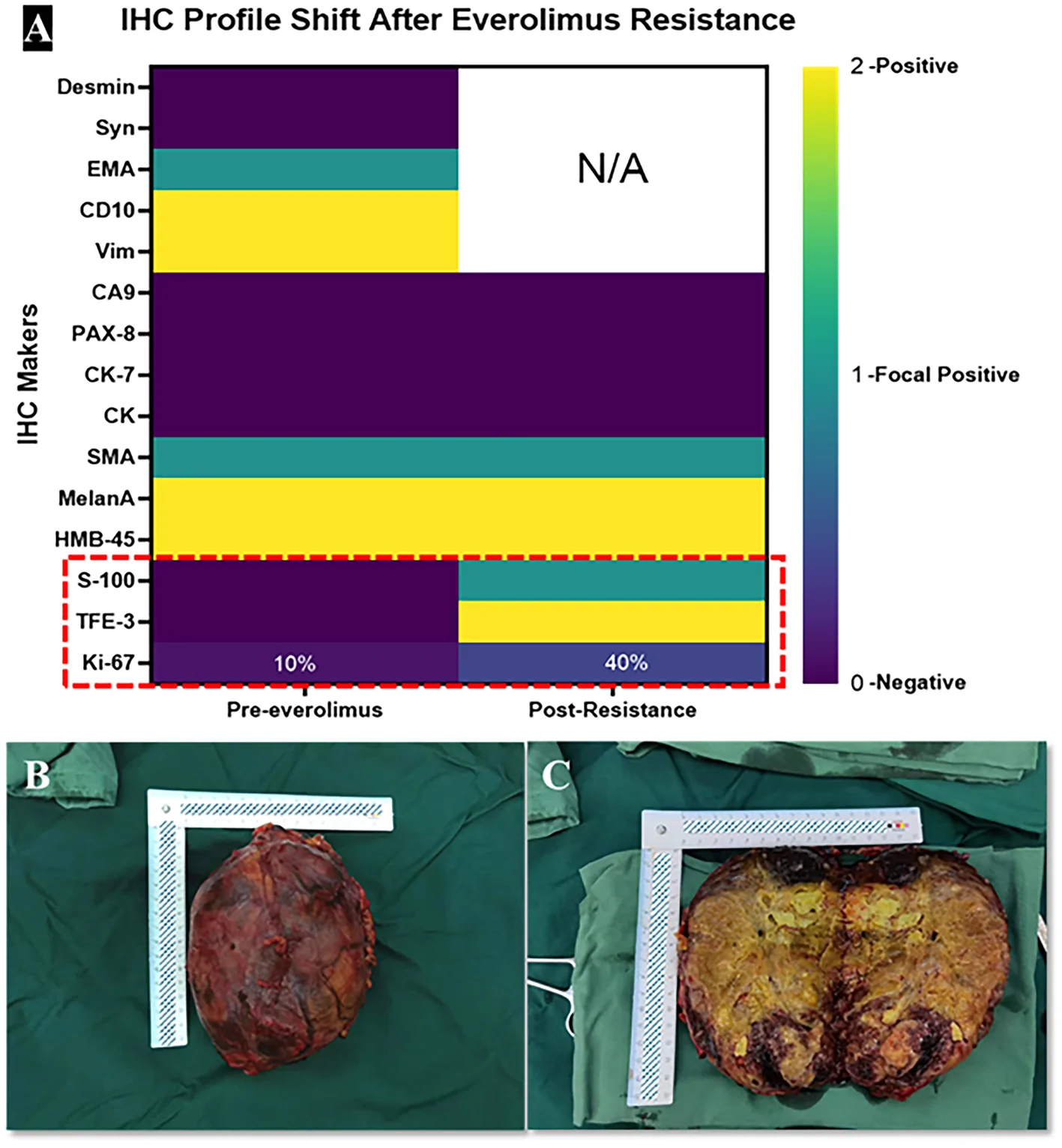
Gross and immunohistochemical features of the EAML. **(A)** Immunohistochemical (IHC) staining of the tumor before and after development of resistance to everolimus. **(B, C)** Gross photographs of the resected left partial nephrectomy specimen. **(B)**Intact specimen demonstrating the well-circumscribed renal mass. **(C)** Cross-sectional specimen showing a solid, heterogeneous cut surface. In the post-resistance specimen, S-100 staining was focally positive (~10% of tumor cells).

Preoperative staging classified the tumor as T2b. Serum tumor markers, including AFP, CEA, CYFRA21-1, NSE, CA125, CA242, and HCG, were within normal ranges (Supplementary Table 1). The patient subsequently underwent open left partial nephrectomy (Figures 2B, C). Final pathological examination confirmed EAML. Immunohistochemistry demonstrated positivity for HMB-45 and Melan-A, focal positivity for SMA, and negativity for S-100 and TFE-3. The Ki-67 proliferation index was approximately 10% (Table 1). Postoperative CT imaging confirmed complete resection with negative margins and no residual lesion (Figures 1D–F).

**Table 1:** Longitudinal comparison of immunohistochemical profiles from initial diagnosis to acquired everolimus resistance.

Marker	Post-operation	Pre-everolimus	Post-resistance	Change
Desmin	–	–	N/A	N/A
Syn	–	–	N/A	N/A
EMA	Focal +	Focal +	N/A	N/A
CD10	+	+	N/A	N/A
Vim	+	+	N/A	N/A
CA-9	–	–	–	N
PAX-8	–	–	–	N
CK-7	–	–	–	N
CK	–	–	–	N
SMA	+	+	+	N
Melan-A	+	+	+	N
HMB-45	+	+	+	N
**TFE-3**	**–**	**–**	**+**	**Y**
**Ki-67**	**10%**	**10%**	**40%**	**Y**
**S-100**	**–**	**–**	**Focal** **+** **(~10%)**	**Y**

IHC, Immunohistochemistry; CK, Cytokeratin; CK-7, Cytokeratin 7; Vim, Vimentin; PAX-8, Paired Box Gene 8; CA-9, Carbonic Anhydrase IX; EMA, Epithelial Membrane Antigen; CD10, Cluster of Differentiation 10; TFE-3, Transcription Factor E3; HMB-45, Human Melanoma Black 45; Melan-A, Melanoma Antigen; S-100, S100 Protein; SMA, Smooth Muscle Actin; Syn, Synaptophysin; Desmin, Desmin; Ki-67, Proliferation Index; –, Negative; +, Positive; Focal +, Focal Positive; N/A, Not Assessed; N, No change; Y, Change. Boldface indicates immunohistochemical markers showing changes in expression after acquired resistance.

### Recurrence and molecular-targeted therapy

**Figure 3:**
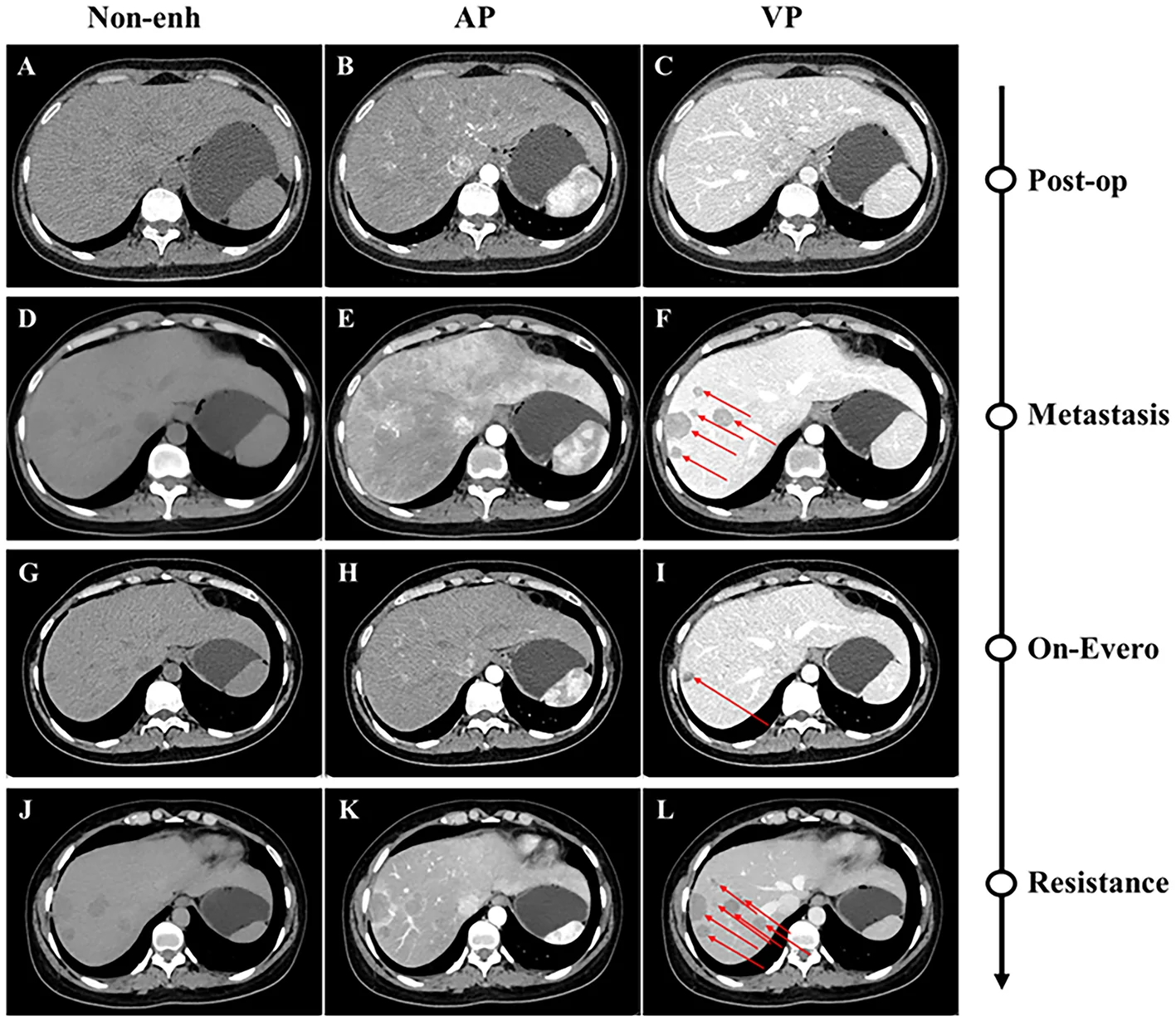
Serial hepatic imaging demonstrates the clinical course from metastatic disease to treatment response and subsequent acquired resistance. **(A–C)** Baseline contrast-enhanced CT images of the liver prior to the development of metastases. **(D–F)** Surveillance CT at 10 months post-surgery showing new hypodense hepatic metastatic lesions (arrows). **(G–I)** Follow-up CT after initiation of everolimus, demonstrating complete radiographic resolution of metastatic lesions. **(J–L)** CT imaging at 16 months of therapy showing disease progression with new hepatic metastases (arrows), consistent with acquired resistance. AP, arterial phase; VP, venous phase; Non-enh, non-enhanced.

Approximately 10 months after surgery, surveillance imaging revealed local recurrence and new hepatic metastases (Figures 1G–I, 3A–F, Supplementary Figures 2A–F and Supplementary Figures 3A–F). A repeat biopsy was performed prior to initiation of everolimus. Histopathological examination confirmed metastatic EAML. Importantly, the immunophenotype remained consistent with the primary tumor (Figure 2A, Supplementary Figures 6A–D and Table 1). Targeted genetic profiling identified biallelic TSC2 mutations (p.R505^*^, exon 15; p.K561^*^, exon 16) and a low tumor mutational burden (TMB, 2.11mutations/Mb). Based on this molecular profile, everolimus (10 mg once daily, orally) was initiated, resulting in a complete radiographic response (CR, Figures 1J–L, 3G–I). Treatment-related adverse events included rash, oral mucositis, and headache, all grade 1–2 and manageable. No grade 3–4 toxicities occurred, and no dose reduction or interruption was required.

### Development of resistance and further management

After 16 months of everolimus therapy, radiological progression was confirmed, consistent with acquired resistance (Figures 1M–O, 3J–K, Supplementary Figures 4A–F and Supplementary Figures 5A–F). A repeat biopsy and molecular analysis demonstrated persistent biallelic TSC2 mutations, along with newly identified homozygous deletions of CDKN2A and CDKN2B at chromosome 9p21.3. The TMB remained low (2.11 mutations/Mb). Histologically, the tumor showed increased proliferative activity, with Ki-67 index rising to approximately 40%. Notably, immunohistochemistry showed focal S-100 positivity (~10% of tumor cells), which was absent in the primary tumor, together with TFE-3 immunoreactivity in a subset of tumor cells. These changes may be related to intratumoral heterogeneity or clonal selection rather than definitive lineage transformation (Figure 2A, Supplementary Figures 6E–H and Table 1). Although CDKN2A/B loss temporally coincided with the hyperproliferative phenotype, a causal relationship cannot be established based on this single observation. Subsequent therapies, including transarterial chemoembolization (TACE), pembrolizumab, axitinib, and anlotinib, were administered based on individualized clinical decision-making; however, no meaningful clinical response was observed. Cytotoxic chemotherapy was considered but was not pursued because of the patient's poor general condition after disease progression and personal preference.

### Outcome

**Figure 4:**
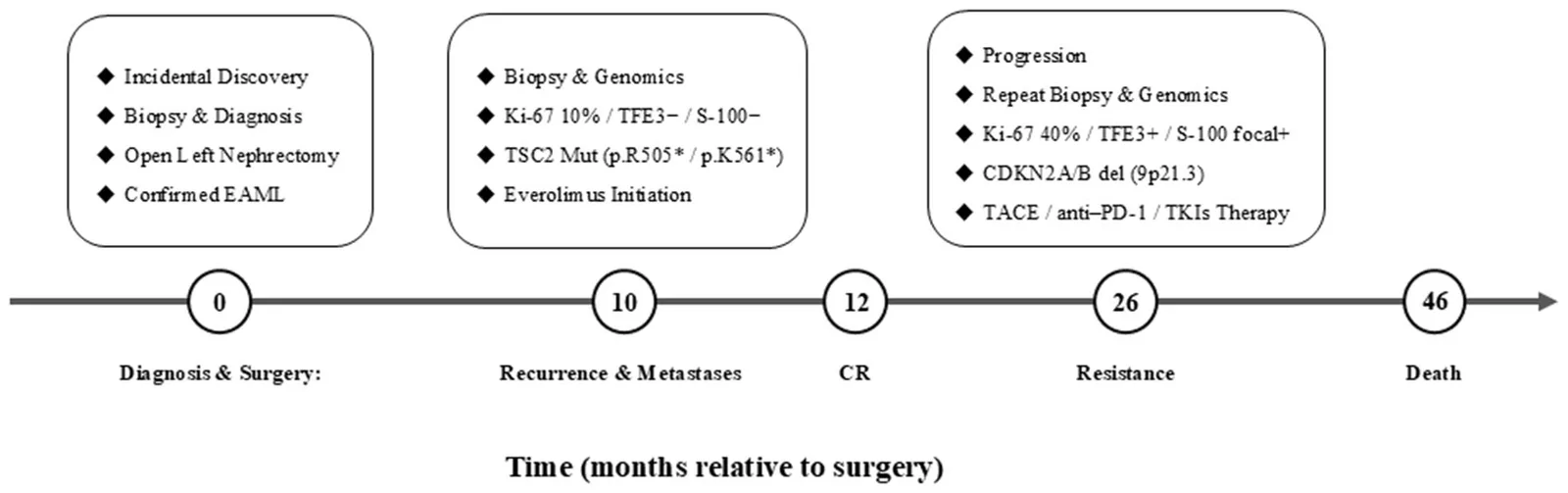
Summarized timeline of the clinical course and molecular evolution. The schematic illustrates key events from initial diagnosis to final outcome, together with corresponding pathological and molecular findings. Major interventions include surgical resection, initiation of everolimus, and subsequent therapies (TACE, pembrolizumab, and TKIs). Molecular findings from serial biopsies are annotated, highlighting the biallelic TSC2 mutations and the subsequent acquisition of CDKN2A/B homozygous deletion. This genetic evolution was accompanied by an increase Ki-67 proliferation index, as well as emergent TFE-3 expression and focal S-100 positivity (~10% of tumor cells) during disease progression. EAML, epithelioid angiomyolipoma; TACE, transarterial chemoembolization; TKI, tyrosine kinase inhibitor; CR, complete radiological response; Mut, mutation.

The patient experienced progressive disease despite multiple lines of therapy and died approximately 46 months after initial surgery (36 months after initiation of everolimus). A summarized timeline of the clinical course and molecular evolution is presented in Figure 4.

## Discussion

This case describes a young patient with metastatic EAML who exhibited an initial marked response to the mTOR inhibitor everolimus, followed by the development of acquired resistance and ultimately fatal disease progression. Longitudinal molecular and histopathological data provide insights into tumor evolution under therapeutic pressure.

The diagnosis of EAML was supported by its characteristic histomorphology and immunophenotype, including positivity for HMB-45 and Melan-A, which helped distinguish it from renal neoplasms, particularly renal cell carcinoma (7, 8). Importantly, identification of biallelic TSC2 mutations (p.R505^*^ and p.K561^*^) indicated constitutive activation of the mTOR pathway, providing a molecular rationale for treatment with everolimus (6, 13). The observed complete radiological response further supports the sensitivity of TSC-mutant PEComa to mTOR inhibition and is consistent with previous clinical reports (14).

The development of acquired resistance remains the major clinical challenge in this disease. Serial biopsy analyses in this case suggest a complex pattern of tumor evolution under therapeutic pressure. The expression of TFE-3 and focal S-100 positivity suggests phenotypic evolution toward a more aggressive tumor phenotype, and its clinical significance requires further investigation. More notably, we identified acquired homozygous deletions of CDKN2A and CDKN2B at 9p21.3. These genes encode key regulators of the G1–S cell cycle checkpoint, including p16INK4a, p14ARF and p15INK4B, and their loss is known to promote cell cycle progression in multiple malignancies (15–18). In this context, CDKN2A/B loss may have contributed to the observed increase in proliferative activity, as reflected by the rise in Ki-67 from 10% to 40%.We propose that persistent mTOR pathway activation driven by TSC2 mutations, together with cell cycle deregulation due to CDKN2A/B loss, likely cooperated in shaping an aggressive and therapy-resistant tumor phenotype. TP53 alteration was not detected in the resistance specimen by genomic profiling. However, given the recognized role of TP53 dysfunction in aggressive sarcomas, its potential contribution cannot be completely excluded. A schematic model summarizing the proposed molecular evolution associated with everolimus resistance is provided in Supplementary Figure 7.

Previous studies have explored mechanisms of resistance to mTOR inhibitors in different tumor types (19). In particular, alterations in the CDK4/6–RB axis and CDKN2A loss have been implicated in reduced sensitivity to rapalogs in preclinical models (20). In addition, activation of PI3K–AKT–mTOR pathway components such as PIK3CA or alterations in mTOR complex regulation (e.g., RICTOR) have also been reported, suggesting that CDKN2A/B loss may represent one component of a broader resistance network (19, 21, 22). Given the rarity of EAML, data on resistance mechanisms remains limited, and whether these alterations are primary drivers or secondary events remains to be clarified.

From a clinical perspective, this case underscores the lack of an established treatment strategy for advanced EAML after mTOR inhibitor resistance. TACE was used for locoregional control of hepatic progression, whereas pembrolizumab was selected as off-label salvage therapy because no standard systemic option was available and occasional responses to PD-1 blockade had been reported in PEComa (23–26). Its failure cannot be attributed to low TMB alone, particularly because MSI/MMR status, PD-L1 expression, and immune infiltration were not assessed (27–29). Cytotoxic chemotherapy was considered but not pursued because the patient had an ECOG performance status of 2, and its anticipated toxicity was judged unfavorable relative to the modest activity reported for anthracycline- or gemcitabine-based regimens in advanced PEComa (10, 30). Subsequent axitinib and anlotinib also failed to achieve meaningful disease control. Although combined mTOR and CDK4/6 inhibition are biologically rational, its efficacy in EAML remains unproven and requires disease-specific validation (31–36).

Tumor heterogeneity and tissue-specific context likely influence the biological behavior of EAML and its response to therapy. Although CDKN2A/B alterations have been associated with mTOR pathway modulation in other tumor types, whether mTOR inhibition can directly promote such genomic alterations remains unclear. Further studies are needed to clarify these evolutionary dynamics in rare mesenchymal tumors such as EAML.

In summary, this case of TSC2-mutant metastasis EAML illustrates both the therapeutic potential and limitations of mTOR inhibition. It highlights the importance of repeat molecular profiling at disease progression to better guide subsequent treatment strategies. The co-occurrence of CDKN2A/B loss, increased Ki-67 index, and histological evolution suggests a potential association between cell cycle deregulation and therapeutic resistance, although causality cannot be established. Combination strategies targeting both mTOR signaling and cell cycle pathways, including CDK4/6 inhibition, warrant further investigation in this rare tumor type.

### Limitations

This study has several limitations. As a single-case report, it cannot determine whether CDKN2A/B deletion is a driver of resistance or a concurrent event during tumor progression. Accordingly, CDKN2A/B loss should be interpreted as being associated with, rather than causative of, treatment resistance. No functional experiments were performed to validate its biological role in mTOR inhibitor resistance. In addition, immune profiling, including PD-L1 expression and tumor-infiltrating lymphocytes, was not performed, limiting interpretation of the immunotherapy response. Therefore, all mechanistic interpretations remain hypothesis-generating.

## Data Availability

The original contributions presented in the study are included in the article/Supplementary material, further inquiries can be directed to the corresponding authors.
