# Supplementary material for: Case Report: Acquired CDKN2A/B deletion and mTOR inhibitor resistance in TSC2-mutant metastatic epithelioid angiomyolipoma
Source: Front Med (Lausanne). 2026 Aug 25;13:1832914. doi: 10.3389/fmed.2026.1832914 (PMC13550966; doi:10.3389/fmed.2026.1832914)
Supplement: Supplementary file 1 [file Supplementary_file_1.docx]

**Supplementary Figure 1. Preoperative multiplanar CT characterization of the primary left renal epithelioid angiomyolipoma.** (A–C) Coronal reformatted contrast-enhanced CT images: (A) non-enhanced, (B) arterial phase, and (C) venous phase, showcasing the heterogeneous renal mass (arrows). (D–F) Sagittal reformatted contrast-enhanced CT images: (D) non-enhanced, (E) arterial phase, and (F) venous phase, further delineating the tumor's anatomical extent and enhancement features (arrows).

**Supplementary Figure 2. Cross-sectional imaging confirms local recurrence at the nephrectomy site.** (A-C) Coronal reformatted contrast-enhanced CT images obtained at recurrence: (A) non-enhanced, (B) arterial phase, and (C) venous phase, demonstrating the recurrent soft tissue mass (arrows). (D-F) Sagittal reformatted CT images in the same patient: (D) non-enhanced, (E) arterial phase, and (F) venous phase, further delineating the extent of the recurrent tumor (arrows).

**Supplementary Figure 3. Detection of newly developed hepatic metastases on surveillance imaging.** (A–C) Coronal reformatted contrast-enhanced CT images: (A) non-enhanced, (B) arterial phase, and (C) venous phase, revealing new hypodense metastatic lesions (arrows) in the liver. (D–F) Sagittal reformatted CT images in the same sequence: (D) non-enhanced, (E) arterial phase, and (F) venous phase, further characterizing the metastatic deposits (arrows) and their enhancement patterns.

**Supplementary Figure 4. Abdominal CT findings upon development of acquired resistance to everolimus.** (A-C) Coronal reformatted contrast-enhanced CT images: (A) non-enhanced, (B) arterial phase, and (C) venous phase, demonstrating progressive disease at the primary site (arrows). (D-F) Sagittal reformatted CT images: (D) non-enhanced, (E) arterial phase, and (F) venous phase, further characterizing the treatment-resistant tumor foci (arrows).

**Supplementary Figure 5. Hepatic metastatic progression during everolimus resistance.** (A-C) Coronal reformatted contrast-enhanced CT images: (A) non-enhanced, (B) arterial phase, and (C) venous phase, revealing new hepatic metastatic lesions (arrows). (D-F) Sagittal reformatted CT images: (D) non-enhanced, (E) arterial phase, and (F) venous phase, confirming the presence and enhancement pattern of liver metastases (arrows).

**Supplementary Figure 6. Immunohistochemical (IHC) and histologic comparison of pre- and post-resistance tumor specimens (×200).** (A–D) Primary tumor sample prior to everolimus treatment: (A) Hematoxylin and eosin (H&E) staining; (B) S-100 staining; (C) TFE-3 staining (negative); (D) Ki-67 immunostaining (index 10%). (E–H) Resistant tumor sample following everolimus therapy: (E) H&E staining; (F) S-100 staining (focal positivity); (G) TFE-3 staining (positive); (H) Ki-67 immunostaining (index 40%). The comparative IHC profiles illustrate the phenotypic evolution toward a more aggressive state upon acquisition of resistance.

**Supplementary Figure 7. Proposed model of molecular evolution during acquired resistance to everolimus in TSC2-mutant EAML.** Schematic representation of the molecular changes observed before and after acquired resistance to everolimus, including persistent TSC2 alterations, CDKN2A/B deletion, increased proliferative activity, and TFE3 immunoreactivity. This model is hypothesis-generating, and dashed lines indicate proposed or correlative relationships.
