# Supplementary material for: Case Report: Acquired CDKN2A/B deletion and mTOR inhibitor resistance in TSC2-mutant metastatic epithelioid angiomyolipoma
Source: Front Med (Lausanne). 2026 Aug 25;13:1832914. doi: 10.3389/fmed.2026.1832914 (PMC13550966; doi:10.3389/fmed.2026.1832914)
Supplement: Supplementary file 2 [file Table_1.docx]

**Supplementary Table 1. Serum tumor marker profile at initial diagnosis.**

| **Serum Marker** | **Measured value** | **Reference range** | **Interpretation** |
| --- | --- | --- | --- |
| AFP (µg/L) | 1.66 | 0-20 | Within reference range |
| CYFRA21-1 (µg/L) | 1.06 | 0-5 | Within reference range |
| CEA (ng/mL) | 0.58 | 0-5 | Within reference range |
| NSE (ng/mL) | 20.28 | 0-25 | Within reference range |
| CA125 (kU/L) | 9.22 | 0-35 | Within reference range |
| CA242 (kU/L) | 3.19 | 0-20 | Within reference range |
| HCG (µg/L) | 0.13 | 0-4 | Within reference range |

Abbreviations: AFP, Alpha-Fetoprotein; CEA, Carcinoembryonic Antigen; CYFRA 21-1, Cytokeratin 19 Fragment; NSE, Neuron-Specific Enolase; CA, Cancer Antigen; HCG, Human Chorionic Gonadotropin. Note: Reference ranges are institution specific.
